# Growth Factor Proteins and Treatment-Resistant Depression: A Place on the Path to Precision

**DOI:** 10.3389/fpsyt.2018.00386

**Published:** 2018-08-23

**Authors:** Alice Pisoni, Rebecca Strawbridge, John Hodsoll, Timothy R. Powell, Gerome Breen, Stephani Hatch, Matthew Hotopf, Allan H. Young, Anthony J. Cleare

**Affiliations:** ^1^Department of Psychological Medicine, Institute of Psychiatry, Psychology and Neuroscience, King's College London, London, United Kingdom; ^2^Department of Biostatistics, Institute of Psychiatry, Psychology and Neuroscience, King's College London, London, United Kingdom; ^3^Social, Genetic and Developmental Psychiatry Centre, Institute of Psychiatry, Psychology and Neuroscience, King's College London, London, United Kingdom; ^4^South London and Maudsley NHS Foundation Trust, London, United Kingdom

**Keywords:** depression, neurogenesis, growth factor, brain derived neurotrophic factor, treatment-resistant depression, biomarker, precision medicine

## Abstract

**Background:** Since the neurotrophic hypothesis of depression was formulated, conflicting results have been reported regarding the role of growth factor proteins in depressed patients, including whether there are state or trait alterations found in patients compared to controls and whether they represent predictors of treatment response. Recently it has been hypothesized that heterogeneity of findings within this literature might be partly explained by participants' history of treatment-resistant depression. This study aimed to investigate the role of growth factor proteins in patients with treatment-resistant depression (TRD) undergoing an inpatient intervention.

**Methods:** Blood samples were collected from 36 patients with TRD and 36 matched controls. Patients were assessed both at admission and discharge from a specialist inpatient program. We examined serum biomarker differences between patients and non-depressed matched controls, longitudinal changes after inpatient treatment and relationship to clinical outcomes. Additionally, the influence of potential covariates on biomarker levels were assessed.

**Results:** Patients displayed lower serum levels of brain-derived neurotrophic factor (OR = 0.025; 95% CI = 0.001, 0.500) and vascular endothelial growth factor-C (VEGFC; OR = 0.083, 95% CI = 0.008, 0.839) as well as higher angiopoietin-1 receptor (Tie2; OR = 2.651, 95% CI = 1.325, 5.303) compared to controls. Patients were stratified into responders (56%) and non-responders (44%). Lower VEGFD levels at admission predicted subsequent non-response (OR = 4.817, 95% CI = 1.247, 11.674). During treatment, non-responders showed a decrease in VEGF and VEGFC levels, while responders showed no significant changes.

**Conclusion:** TRD patients demonstrate a deficit of peripheral growth factors and our results suggest that markers of the VEGF family might decline over time in chronically depressed patients in spite of multidisciplinary treatment. The action of angiogenic proteins may play an important role in the pathophysiology of TRD, and pending comprehensive investigation may provide important insights for the future of precision psychiatry.

## Introduction

Major Depressive Disorder (MDD) is now considered the leading cause of disability worldwide (1). Understanding the pathophysiology of this disorder is essential to optimizing treatment, however the underlying neurobiological mechanisms are still not fully understood. The neurotrophic and neurogenic hypothesis of depression (2) postulates that stress-induced alterations in neurotrophic action mediate reduced adult neurogenesis and volume reductions in the hippocampus which ultimately increase risk for mood disorders (3). Antidepressant use is hypothesized to reverse this process and increase the proliferation of progenitor cells by stimulating the production of growth factors, molecules responsible for neurogenesis and maintenance of neural networks (4, 5).

Evidence for a role of growth factors in the pathophysiology of depression has come from clinical studies mainly investigating brain derived neurotrophic factor (BDNF), a neurotrophin involved in processes of neuronal maturation, synapse formation and synaptic plasticity (3), and vascular endothelial growth factor (VEGF or VEGFA), an angiogenic factor also possessing neurotrophic and neuroprotective properties (6, 7).

Research has reported lower BDNF levels in post-mortem brains of depressed patients compared to non-depressed controls (8–10), although these appear to be higher in those patients who had taken antidepressants (11). Low levels of BDNF have also been found in the blood of depressed patients, with increases reported following antidepressant treatment (12–14).

On the contrary, levels of VEGF tend to be elevated in depressed patients (15, 16), although a number of studies have reported no significant differences compared with non-depressed controls (see 17 for a review). The effects of antidepressants on VEGF are also not clear-cut, with some studies reporting no changes (18–21), one reporting a decrease (22) and one reporting an increase correlated with improvement of depressive symptomatology (23).

Recently, resistance to treatment has been suggested as a potential confounding factor in this field of research (17). Treatment-resistant depression (TRD) is common and contributes substantially to the burden of depression (24). More pronounced reductions of proteins central to cellular growth and proliferation might be expected in patients with TRD, which could be a risk factor and/or consequence of an unsuccessfully treated affective illness. Indeed, limited research has found lower BDNF levels in TRD than both non-depressed controls and treatment-responsive patients (25). Measuring a similar cohort to the present study, Carvalho et al. identified non-significantly lower VEGF levels in participants with TRD who did not go on to respond to an inpatient treatment package than responder participants [*p* = 0.058; (26)].

Research to date has not identified sufficiently consistent effects to progress the pathway toward precision medicine, perhaps in part due to studying heterogeneous depressed groups and limited trophic biomarker panels. We aimed to address these drawbacks by examining a severe TRD population (alongside non-depressed, matched controls) and monitoring them during a naturalistic course of inpatient treatment in addition to a long-term follow-up. Alongside the well-researched BDNF and VEGF, we also considered six growth factors that play a role in neurogenesis and maintenance of neural connections but have never been investigated in TRD; due to the scant evidence in our possession surrounding their role in depression, these comparisons were exploratory in nature. We therefore test three main two-tailed hypotheses: First, that patients and controls would differ in levels of growth factors; second, that growth factor levels would change between pre- and post-treatment assessments; and third, that protein levels would differ between subsequent responders and non-responders to inpatient treatment.

## Materials and methods

This study was approved by the Camberwell & St. Giles NHS Research Ethics Committee (TRD patients; reference 322/03) and King's College London Research Ethics Committee (non-depressed controls; reference PNM/12/13-152). In accordance with the recommendations of the Declaration of Helsinki, all participants provided written informed consent prior to participation.

### Participants

#### TRD patients

A cohort of 36 TRD patients were naturalistically recruited and treated within a specialist inpatient unit for treatment-resistant mood disorders (National Affective Disorders Unit, South London and Maudsley NHS Foundation Trust, UK). Assessments took place as close as possible after admission, and before discharge; mean treatment duration 6 months. Patients met inclusion criteria if they had a primary diagnosis of an affective disorder (unipolar or bipolar) and were currently depressed, defined as a score ≥8 using the Hamilton Depression Rating Scale [HDRS-17; (27)]. The diagnosis was defined following DSM-IV and ICD-10 criteria, assessed using the Mini International Neuropsychiatric Interview [MINI; (28)] and confirmed by two psychiatrists and a screening of patients' records. Upon admission, all patients were treatment-resistant, defined by a score >7.5 using the Maudsley Staging Method [MSM; (29)], and taking medications. Patients underwent a multidisciplinary intervention, including pharmacological, psychological and occupational treatment, as well as electroconvulsive therapy (ECT) in some cases, however not all participants underwent all types of treatment. All patients completed non-biological measures at both time points, and blood collection at admission; 7 patients were unavailable for venepuncture measurement at the discharge time point.

#### Control participants

36 non-depressed controls were selected from the South East London Community Health study (SELCoH) based on closeness of matching to the TRD sample in age, gender and BMI [see (30) for more information regarding the SELCoH study]. Control participants did not meet criteria for current psychiatric disorders measured using the Clinical Interview Schedule-Revised (31) and did not have significant depressive symptoms as indicated by a score <10 on the Patient Health Questionnaire (32).

### Measures

#### Biomarkers

Levels of eight different biomarkers were measured: angiopoietin-1 receptor (Tie2), brain-derived neurotrophic factor (BDNF), vascular endothelial growth factor (VEGF), vascular endothelial growth factor-C (VEGFC), vascular endothelial growth factor-D (VEGFD), placental growth factor (PlGF), basic fibroblast growth factor (bFGF), and soluble fms-like tyrosine kinase-1 (sFlt1; also termed soluble VEGF receptor-1). Blood for serum samples (1 × 5ml tube) was collected in the morning between 9 and 11 a.m. Following complete clotting, the tubes were centrifuged and serum extracted, transferred into cryovials and frozen (between −40° and −80°C). Serum concentrations of biomarkers were assayed in duplicate with ultra-high sensitivity Meso Scale Discovery (MSD) V-plex kits (Meso Scale Diagnostics, Maryland, USA), shown to be a reliable measurement tool (33). Unless otherwise stated, protein levels are reported in pg/ml.

#### Non-biological assessments

These were conducted in the TRD group only. Depression severity was measured using a clinician-administered rating scale [HDRS-17; (27)], with treatment response defined as more than 50% reduction in scores between admission and discharge time points. Severity of treatment resistance was assessed at admission using the Maudsley Staging Method [MSM; (29)]. History of childhood adversity was measured using the Childhood Trauma Questionnaire [CTQ; (34)]. Cognitive impairment was assessed at admission using the Mini-Mental State Examination [MMSE; (35)]. Physical health was assessed at admission using the Modified Cumulative Illness Rating Scale [MCIRS; (36)], with the total score calculated excluding the item pertaining to mental health. Demographic data was obtained at admission. Number of medications were recorded at each time point, and changes during treatment were noted at discharge.

### Statistical analyses

Raw biomarker data was standardized using logarithmic transformation (base log10) before undergoing analyses. All data analyses were carried out using bootstrapping, with 1000 generated samples.

#### Primary analyses

Logistic regressions and paired *t*-tests were used to test the primary null hypotheses, testing differences between responders and non-responders. Conditional logistic regressions compared the differences in biomarker levels between individually matched TRD patients and controls at each time point, accounting for gender, age and BMI. Other covariates were individuated using correlational analyses (see below; secondary analyses) and added to the relevant regression model if both correlations and unadjusted analyses were significant. Repeated measures ANOVAs were performed to identify changes in biomarker levels after treatment, using time as the within-subject variable.

Due to the number of comparisons, a False Discovery Rate (FDR) control for multiple testing was applied to primary analyses to reduce the probability of type I error. Thus, uncorrected *p* values < 0.05 are reported as tentatively significant findings and *q* values < 0.1 as significant (37).

#### Secondary analyses

Paired sample *t*-tests were performed to examine longitudinal changes in the responder and non-responder groups individually. Pearson's correlations tested for possible association between different biomarkers, as well as between biomarkers and potential covariates in the TRD group, namely depression severity at admission and discharge, childhood trauma, cognitive impairment, physical health, severity of treatment-resistance, number of medications, and number of medication changes during inpatient treatment (i.e., starting or stopping an antidepressant medication, but not changes in dosage).

## Results

### Sample characteristics

There was a preponderance of female participants (*n* = 42; male = 30). Mean age at admission was 54.54 (SD = 13.85). Mean BMI was 28.19 (SD = 5.16). Descriptive statistics for demographic and biomarker data can be found in Table 1. 20 patients (55.6%) were classified as responders, and 16 patients as non-responders (44.4%). The two subgroups did not differ in other clinical or sociodemographic factors. Mean values for all measures, including scores from questionnaires, can be found in supplementary material (Supplementary Table 1).

**Table 1:** Participant characteristics.

		**Non-depressed controls (*****n*** = **36)**		**TRD patients (*****n*** = **36)**		***p*-value**
		**Log-mean (SD)**	**Range**	**Log-mean (SD)**	**Range**	
Age		54.48 (13.78)	28–80	54.55 (14.30)	26–83	0.970
BMI		28.11 (4.64)	20.20–41.60	28.22 (5.75)	18.00–46.00	0.930
Biomarker levels (log-pg/ml)						
Pre-treatment	Tie2	3.57 (0.12)	3.32–3.84	3.66 (0.07)	3.38–3.84	0.006^*^
	PlGF	1.44 (0.15)	1.16–1.82	1.43 (0.13)	1.04–1.71	0.547
	VEGF	2.50 (0.30)	1.84–3.05	2.53 (0.41)	1.55–3.31	0.652
	VEGFC	2.55 (0.15)	2.30–2.91	2.45 (0.29)	1.72–2.85	0.045^*^
	VEGFD	2.87 (0.19)	2.29–3.16	2.77 (0.20)	2.21–3.46	0.087
	bFGF	0.70 (0.31)	−0.10–1.23	0.53 (0.63)	−1.22–1.94	0.084
	sFlt1	1.90 (0.13)	1.65–2.30	1.89 (0.12)	1.51–2.17	0.627
	BDNF	4.28 (0.17)	3.68–4.63	4.04 (0.37)	3.02–4.42	0.012^*^
Biomarker levels (log-pg/ml)						
Post-treatment	Tie2			3.68 (0.08)	3.57–3.88	0.010^*^
	PlGF			1.48 (0.08)	1.30–1.64	0.295
	VEGF			2.44 (0.57)	1.57–3.37	0.728
	VEGFC			2.28 (0.48)	1.07–2.80	0.007^*^
	VEGFD			2.79 (0.19)	2.08–3.22	0.196
	bFGF			0.66 (0.53)	−0.39–1.77	0.572
	sFlt1			1.87 (0.13)	1.52–2.10	0.354
	BDNF			4.10 (0.25)	2.98–4.40	0.126

* Different between patients and controls (p < 0.05)

*Other factors did not differ between patient and control groups, as indicated by p-values. For TRD patients as a whole group, no biomarker changes occurred during treatment*.

### Biomarker characteristics

As BDNF levels obtained for one participant in the control group did not reach the lowest limit of detection (LLOD, 30 pg/mL; Meso Scale Diagnostics, Maryland, USA), this datum was initially replaced by half of the LLOD (38), but the dataset became highly skewed, thus this datum was excluded from the dataset. No other biomarker data was outside of detectable limits. Several variables had slightly skewed or kurtotic distributions; where this affected statistical test assumptions, the relevant variable was standardized using *z* scores prior to regression analyses.

### Differences between patients and controls

**Figure 1:**
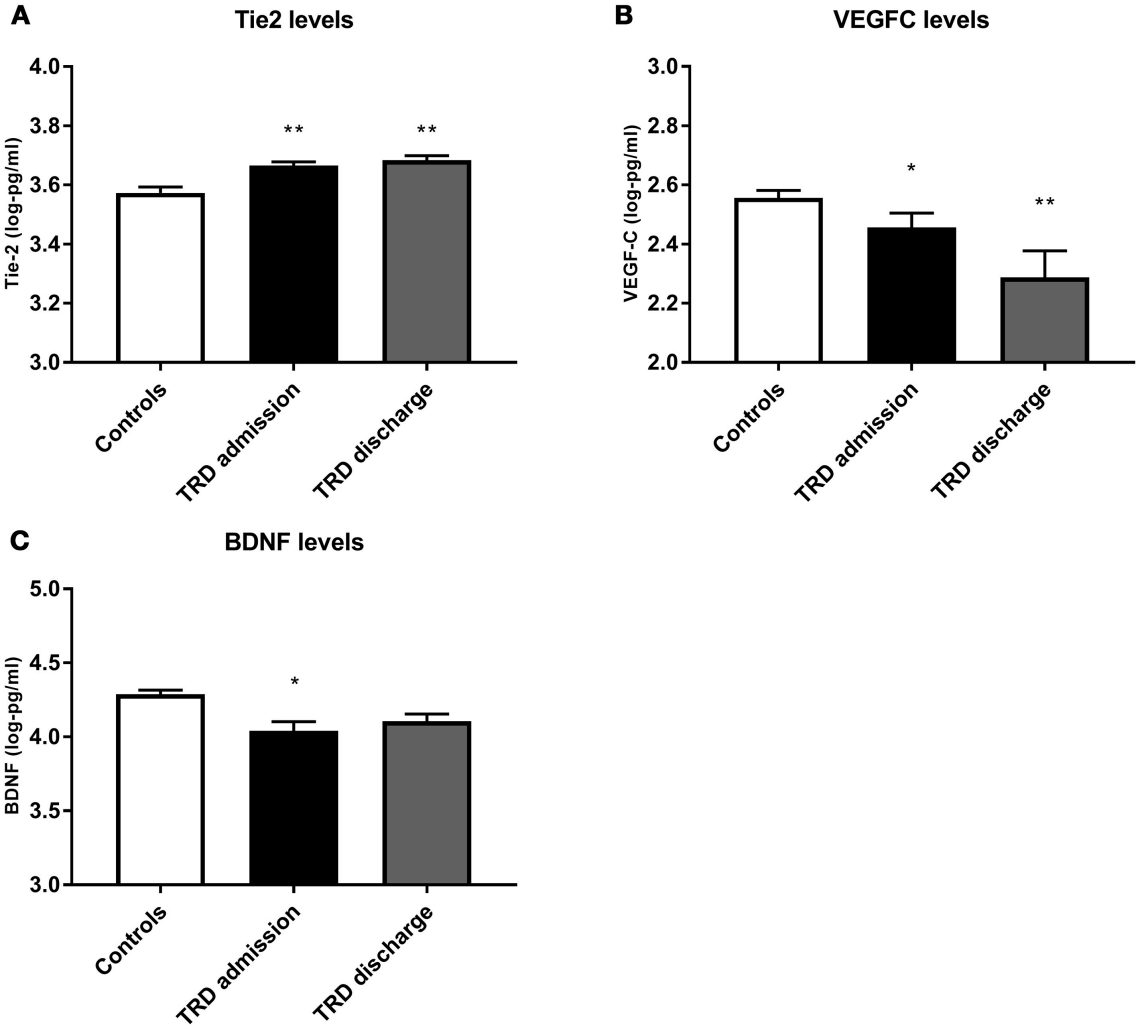
Comparison of protein levels between controls (white bar) and TRD patients on admission (black bar) and discharge (gray bar) from the inpatient unit, for **(A)** Tie2, **(B)** VEGF C and **(C)** BDNF. Error bars show standard error. Note that axes have been cut according to protein levels expressed to clearly depict group differences. *, significantly different from controls at *p* < 0.05. **, significantly different from controls at *p* < 0.01.

Conditional logistic regressions demonstrated three biomarkers as significantly different between the TRD and control groups at both time points. Tie2 was significantly higher in TRD patients [admission: *X*2_(1)_ = 11.67, *p* = 0.006, *q* = 0.048; discharge: *X*2_(1)_ = 11.82, *p* = 0.010, *q* = 0.070]. VEGFC was significantly lower in the TRD group [admission: *X*2_(1)_ = 3.33, *p* = 0.045, *q* = 0.270; discharge: *X*2_(1)_ = 7.85, *p* = 0.007, *q* = 0.056]. Lower BDNF was also found in TRD participants [admission: *X*2_(1)_ = 11.92, *p* = 0.012, *q* = 0.084) but was not significant at discharge [*X*2_(1)_ = 5.56, *p* = 0.126]. Visual representations of these differences are depicted in Figure 1. Table 2 details the conditional logistic regressions used to compare protein levels between these matched groups of TRD and non-depressed groups. Finally, due to the wide age range we presented scatter plots of BDNF, Tie2 and VEGFC in correlation with age in patients and controls; these were not significantly related (see Supplementary Figure 1).

**Table 2:** Conditional logistic regression of biomarker levels (TRD vs. control group; *N* = 72).

**Biomarker**	**OR**	**X^2^**	**95% Confidence intervals**		***p***
			**Lower**	**Upper**	
**PRE-TREATMENT**					
Tie2	2.651	11.672	1.325	5.303	0.006^*^
PlGF	0.344	0.383	0.011	10.386	0.547
VEGF	1.365	0.216	0.366	5.089	0.652
VEGFC	0.159	3.327	0.018	1.362	0.045^*^
VEGFD	0.118	3.738	0.011	1.220	0.087
bFGF	0.288	2.949	0.124	1.209	0.084
sFlt1	0.377	0.273	0.009	15.098	0.627
BDNF	0.025	11.921	0.001	0.500	0.012^*^
**POST-TREATMENT**					
Tie2	3.008	11.823	1.308	6.917	0.010^*^
PlGF	1.326	1.156	0.781	2.251	0.295
VEGF	0.818	0.123	0.266	2.519	0.728
VEGFC	0.083	7.853	0.008	0.839	0.007^*^
VEGFD	0.128	2.064	0.007	2.475	0.196
bFGF	0.681	0.413	0.209	2.220	0.572
sFlt1	0.201	0.809	0.006	7.007	0.354
BDNF	0.028	5.557	0.001	1.338	0.126

* *Different between patients and controls, at both p < 0.05 and q < 0.1*.

*OR, odds ratio; x^2^, Chi-square*.

*Other factors did not differ between patient (n = 36) and control groups (n = 36) following FDR control for multiple comparisons*.

### Biomarkers as predictors of response

**Figure 2:**
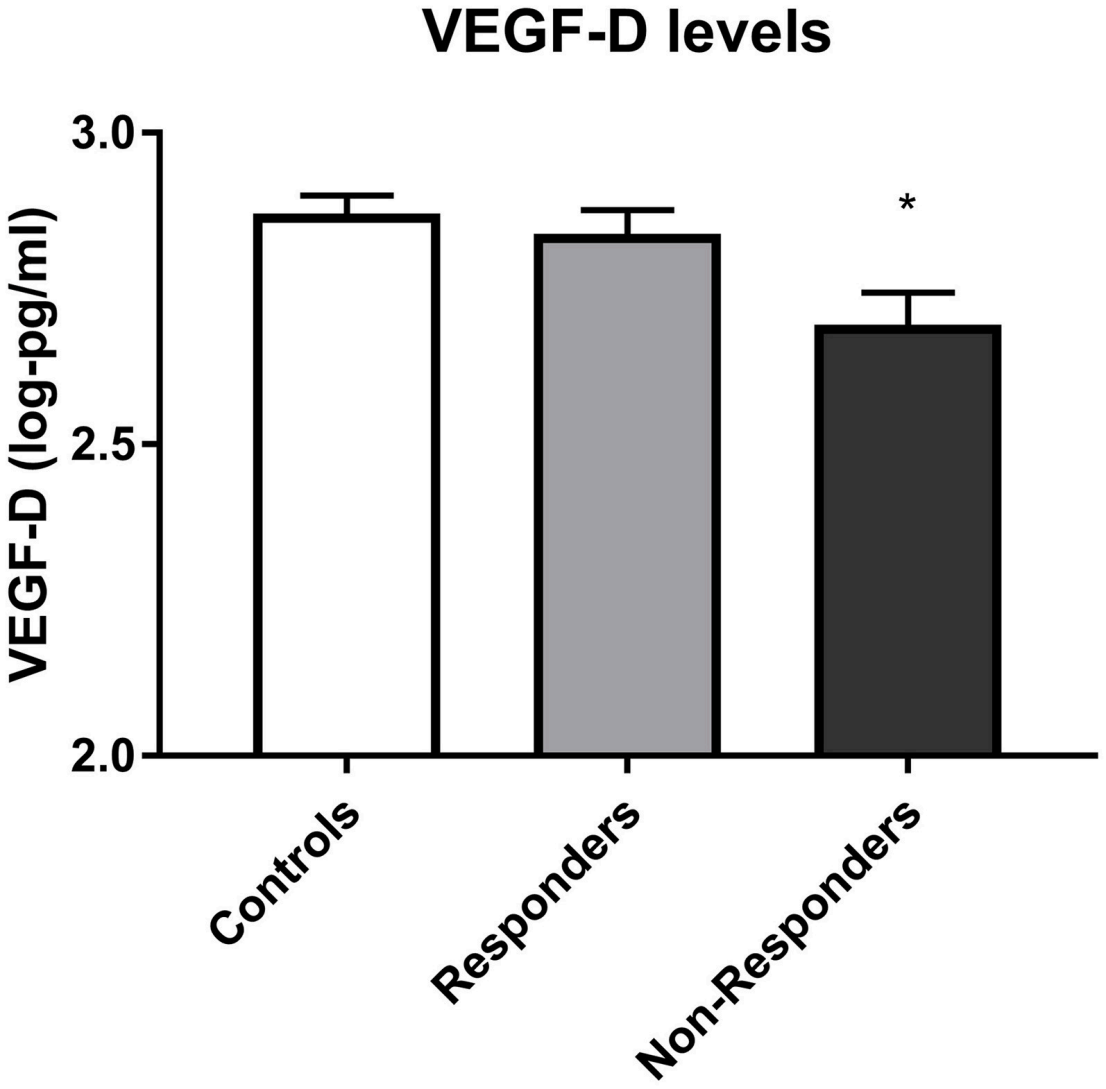
VEGF D levels in controls, responders and non-responders before starting treatment. Error bars show standard error. Note that axes have been cut according to protein levels expressed to depict differences clearly. Lower VEGFD predicted subsequent non-response to treatment (*p* = 0.014). *, subsequent non-responders significantly different from responders at *p* < 0.05.

High admission VEGFD predicted response with tentative significance (responder 2.83 ± 0.17 vs. non-responder 2.69 ± 0.21, respectively), as shown in Figure 2 [*X*2_(1)_ = 5.38, *p* = 0.014, *q* = 0.112]. A trend for higher BDNF levels in responders at admission was also found, however it did not reach significance (*p* = 0.067, *q* = 0.462).

### Changes following inpatient treatment

**Figure 3:**
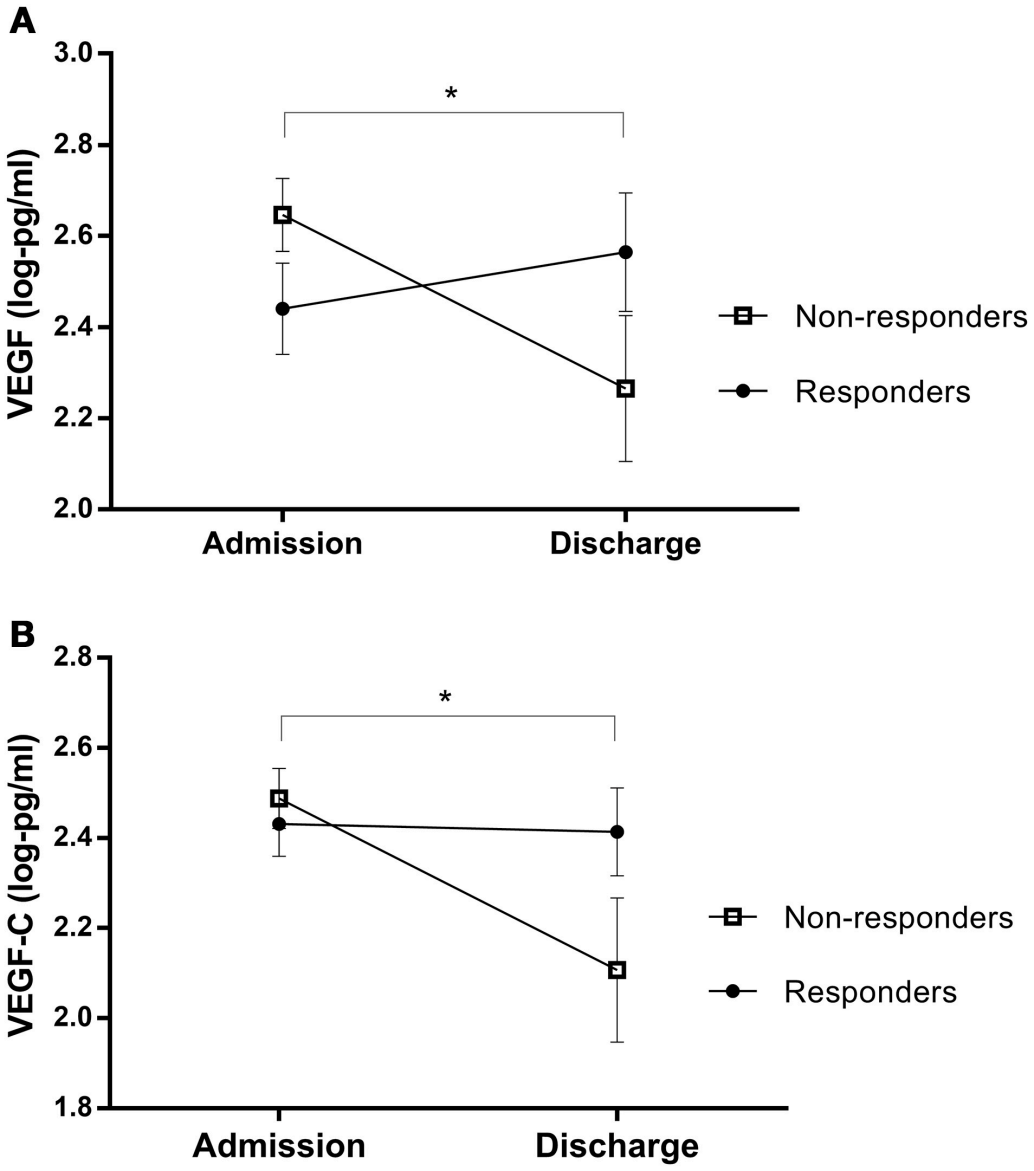
Protein changes in non-responders and responders pre- and post-treatment, for **(A)** VEGF and **(B)** VEGF-C levels. Error bars show standard error. Note that axes have been cut according to protein levels expressed to depict differences clearly. *, The interactions of response status were significant at *p* < 0.05, rather than cross-sectional differences between responder and non-responder patients.

Analyses revealed no significant overall differences between biomarker levels at admission and discharge (pre- and post-treatment protein levels are outlined in Table 1). However, after stratifying based on response, paired samples *t*-tests showed that non-responders experienced a decrease in VEGF and VEGFC during treatment [VEGF: *t*_(11)_ = 2.87 *p* = 0.015, *q* = 0.120; VEGFC: *t*_(11)_ = 2.71, *p* = 0.020, *q* = 0.140], while responders' levels did not change (VEGF: *p* = .491; VEGFC: *p* = .957); see Figure 3.

### Secondary analyses

Two independent samples *t*-tests compared biomarker levels between patients diagnosed with unipolar and bipolar depression, both at inpatient admission and discharge. bFGF levels at admission were higher in unipolar (M = .69, SD = .58) compared to bipolar patients (M = 0.22, SD = 0.65); *t*_(34)_ = 2.19, *p* = 0.038. No significant differences were identified at discharge.

Most biomarkers were inter-correlated, with the exception of BDNF which was not correlated with any other proteins.

Importantly, levels of biomarkers were not associated with depression severity at either time point. Significant correlations were found between biomarkers and other covariates: Tie-2 levels at admission positively correlated with BMI (*r* = 0.46, *p* = 0.024), while admission VEGFC levels were negatively correlated with both BMI (*r* = −0.45, *p* = 0.027) and poorer physical health score (*r* = −0.47, *p* = 0.022). PlGF levels at admission positively correlated with both number of medications (*r* = 0.50, *p* = 0.013) and number of changes in medications that occurred subsequently during treatment (*r* = 0.44, *p* = 0.029). Similarly, bFGF levels at admission positively correlated with number of medications taken (*r* = 0.50, *p* = 0.013) and levels at discharge negatively correlated with number of changes in medication that had taken place since the baseline research assessment (*r* = −0.55, *p* = 0.014). Non-biological variables did not differ between responders and non-responders (see Supplementary Table 1).

## Discussion

The findings from this study may have notable implications for more personalized, predictive approaches to treatment selection for people with depression who have not responded to multiple treatments. Results from the main analyses showed that Tie2 levels were higher in TRD patients than controls, while VEGFC and BDNF were lower in the TRD participants. The BDNF finding replicates two previous clinical studies on TRD (25, 39), which appear to indicate an association between resistance to pharmacological treatment and extremely low levels of BDNF, with implications for the role of neurogenesis and neuroplasticity in therapeutic response. If BDNF expression mediates the action of antidepressants on neural birth and maintenance, patients with low availability of this growth factor may necessitate other forms of therapy in order to elicit a meaningful response. The minimisation of this difference by discharge from this specialist inpatient program support this theory, although we note there were not differences identified between responders and non-responders.

Interestingly, levels of VEGF were not significantly different between patients and controls. Previous work has found levels of VEGF in depressed patients to be either higher than or the same as those found in controls (15–17), and TRD has been proposed as a potential confounder responsible for this heterogeneity. It has been suggested that patients with non-resistant depression display higher VEGF levels, representing a neuroprotective attempt by specific brain structures in response to stress (17). On the other hand, patients with TRD fail to present this automated reaction, preventing response to antidepressants. Our data could support this hypothesis by indicating no difference between VEGF levels in TRD patients and controls, though responders and non-responders also did not differ in VEGF levels.

VEGFC levels were lower in TRD patients than controls. VEGFC has not yet, to our knowledge, been investigated in depression, however it belongs to the same protein family of VEGF, and the two were highly correlated (*p* < 0.001). This could indicate that low availability of growth factors belonging to the VEGF network may be associated with TRD.

Finally, Tie2 was found to be higher in TRD patients compared to controls. Despite the paucity of data surrounding Tie2's function in depression (and absence of data in TRD), this result may be representative of increased inflammatory signaling (40), as would be expected in these patients (41, 42).

After stratifying participants based on treatment response, analyses indicated a decrease of VEGF and VEGFC over time, only in non-responders. Previous studies have found response not to interact with VEGF changes during pharmacological treatment for non-TRD depressed samples (18, 19, 21). Our result could suggest that while an increase in VEGF is not necessary for the therapeutic effects of antidepressants, the non-responders' decrease may represent a progressive loss of neurotrophic action. It is notable here that the treatment period averaged at 6 months, which is of longer duration than the majority of previous research studies within this literature.

No changes were seen in levels of BDNF following antidepressant treatment, contrasting theories that an increase in the availability of this biomarker is a key mechanism in antidepressant action (43). It is likely that these inconsistencies stem in part from heterogeneity of type of treatment, as well as clinical profile and time length between measurement points, as appears to pervade biological research in depression (44). Specifically, all patients were taking multiple pharmacotherapy throughout the admission and for the majority of patients this included mood stabilizer medications, which in this sample were far more frequently taken (27/36 patients) than monoaminergic medications (20/36 patients), although both have been posited to upregulate BDNF (43).

Non-responders displayed significantly lower levels of VEGFD at admission compared to responders. This is, to our knowledge, the first study to examine VEGFD levels in patients with depression, but in addition to its angiogenic and lymphangiogenic functions, this protein also helps to restore and maintain dendritic complexity in the hippocampus (45). Thus, lower levels before antidepressant treatment may have contributed to the reduced clinical benefits for non-responder patients. In similar vein, VEGF has been studied as a potential predictor of antidepressant response, twice in TRD samples. In a recent study by Clark-Raymond et al. (18) involving 38 MDD patients, higher VEGF levels were found in remitters, compared to patients who did not respond to pharmacological therapy. Likewise, Carvalho et al. (26) found a trend for lower levels of VEGF in a small sample of non-responder TRD patients, and Minelli et al. (46) found that lower levels of VEGF predicted lack of response to ECT in a large cohort of 67 TRD patients. In the latter study, VEGF predicted response to ECT but not for another subgroup of MDD patients receiving pharmacological treatment. The authors argue that these results indicate a predictive potential of VEGF specific to TRD. Interestingly, higher levels of VEGF have been found to downregulate the activity of multi-drug resistance transporter at the blood-barrier (47), resulting in increased concentrations of exogenous compounds in the brain, including antidepressants (48). Thus, a greater availability of VEGF may result in larger quantity of antidepressant reaching the brain, while low levels of VEGF in TRD patients (discussed above) may contribute to a low cerebral concentration of antidepressants, insufficient to produce a therapeutic response (46). The authors argue that ECT boosts VEGF availability, thus increasing the effectiveness of antidepressants. However, a challenge to this hypothesis comes from the observation that amelioration of symptoms following ECT is not associated with an increase in VEGF (49). Thus, the temporal relationship between these two events with regards to the blood-barrier hypothesis need to be further investigated, as well as the role played by VEGFD.

### Limitations and future directions

Not all potentially significant results survived the FDR control for multiple comparisons. It may be that the smaller effects of biomarkers predicting response in this study were false positive findings, or that the small sample size and lack of consistently strong inter-correlations between proteins caused these comparisons to be non-significant after FDR control. It is our hope that future studies will help to elucidate this.

The naturalistic approach adopted in this study allowed for an unbiased observation of patients within a realistic clinical environment. The methodological challenges that this presents should be considered when interpreting these findings. Particularly, data on the type of medication prescribed for each patient and which treatments were undertaken during the inpatient program were highly variable and thus challenging to model. The vast majority of TRD participants were undergoing concomitant treatment with antipsychotics and/or mood stabilizers in addition to monoaminergic antidepressants, and such combinations may have unknown and unpredictable effects on growth factor levels. Moreover, data on participation in ECT would have been essential to explore the hypothesis that ECT leads to greater percentage of medication entering the brain following a moderation of the permeability of the blood-barrier by VEGF (46). These issues require further clarification and should be addressed by future studies on TRD. It is also important to consider that growth factor levels are known to fluctuate in response to a number of variables, including food intake (50), exercise and sedentary behavior (51, 52), and even the menstrual cycle (53), representing a common limitation in this type of clinical study. Furthermore, it is important to consider that the TRD and control samples were obtained from two separate studies; as such, differences in sampling conditions may have affected the results. Finally, reduced follow-up biomarker data for patients and the small sample size of our study represent clear limitations. Thus, future research should focus on replicating these findings in larger samples to confirm the importance of the VEGF protein family and Tie2, as factors displaying angiogenic properties have the potential to play a role in the psychopathology of TRD. Furthermore, in order to shed light on potential differences between TRD and non-refractory depression, controlled studies comparing these two clinical groups are desirable, possibly adopting a longitudinal design to monitor changes following discharge. Finally, it has been suggested that our current lack of information on TRD stems mainly from the *post-hoc* design of many studies. To solve this issue, it would be helpful to examine patients at the time of their initial contact with mental health services, and follow them to identify whether biomarker levels represent a predictor of risk of TRD (54).

In conclusion, the present study provides support that compounds such as BDNF and VEGF are important markers in treatment-resistant depression and provides new information on the dynamics of growth factors in TRD. Specifically, longitudinal activity of VEGF-family members might represent candidates for stratifying patients based on likelihood of response. Results have highlighted the importance of angiogenic proteins, which have the potential to represent unique biomarkers of TRD and may be involved in mechanisms of response. Although replication studies in larger samples are needed before definitive conclusions can be drawn, findings from this study characterize novel trophic biomarkers that hold promise as new targets for mood disorder treatment strategies.

## Author contributions

AP contributed to study conception and conducted data analysis, interpretation and writing of the manuscript. RS was involved in the study's conception, data collection, analysis, interpretation, and writing of the manuscript. JH contributed to the design and supervision of statistical analysis and interpretation of data. TP, GB, SH, MH, and AY were involved with study conception and/or design. TP undertook laboratory work and generated the protein data. AC was substantially involved with study conception and design and interpretation of data. Additionally, all authors contributed to drafting/revising the article and approved the final version for publication.

### Conflict of interest statement

AC has in the last 3 years received honoraria for speaking from Astra Zeneca and Lundbeck (AZ), honoraria for consulting from Allergan, Livanova, Janssen and Lundbeck, support for conference attendance from Janssen and research grant support from Lundbeck and UK funding agencies (NIHR, MRC, Wellcome Trust). MH is principal investigator of the RADAR-CNS consortium, a public private precompetitive consortium co-funded by European Commission and members of European Federation of Pharmaceutical Industries and Associations (EFPIA) including Janssen, Lundbeck, Merck, UCB and Biogen. AY has undertaken paid lectures and advisory boards for all major pharmaceutical companies with drugs used in affective and related disorders but has no shareholdings in pharmaceutical companies, has been lead Investigator for the Embolden Study (AZ), the BCI Neuroplasticity Study, and the Aripiprazole Mania Study, investigator-initiated studies from AZ, Eli-Lilly, Lundbeck, and Wyeth, grant funding (past and present) from NIMH (USA), CIHR (Canada), NARSAD (USA), Stanley Medical Research Institute (USA), MRC (UK), Wellcome Trust (UK), the Royal College of Physicians (Edin), BMA (UK), UBC–VGH Foundation (Canada), WEDC (Canada), CCS Depression Research Fund (Canada), MSFHR (Canada), and NIHR (UK). GB has received consultancy fees and funding from Eli Lilly. The authors report no further conflicts of interest in this work.

The remaining authors declare that the research was conducted in the absence of any commercial or financial relationships that could be construed as a potential conflict of interest.
