# Supplementary material for: Growth Factor Proteins and Treatment-Resistant Depression: A Place on the Path to Precision
Source: Front Psychiatry. 2018 Aug 23;9:386. doi: 10.3389/fpsyt.2018.00386 (PMC6115516; doi:10.3389/fpsyt.2018.00386)

Supplementary Material

**Supplementary Table 1: Non-biological questionnaire data**

|  | **Total (n = 36)**  Mean SD | | | **Responders (n = 20)**  Mean SD | | | | **Non-Responders (n = 16)**  Mean SD | | | |  |
| --- | --- | --- | --- | --- | --- | --- | --- | --- | --- | --- | --- | --- |
| Resistance severity (MSM) | 11.9 | 1.8 |  | | 11.6 | 1.7 |  | | 12.1 | 1.8 |  | |
| Depression severity at T1 (HDRS) | 21.9 | 5.6 |  | | 21.1 | 5.2 |  | | 23.2 | 5.8 |  | |
| Depression severity at T2 (HDRS) | 11.9 | 5.7 |  | | 7.5 | 3.4 |  | | 17.9 | 1.8 |  | |
| Childhood trauma (CTQ) | 56.5 | 20.4 |  | | 53.3 | 15.3 |  | | 63.5 | 27.3 |  | |
| Cognitive impairment (MMSE) | 27.2 | 3.3 |  | | 27.3 | 3.3 |  | | 28.0 | 3.2 |  | |
| Physical health (MCIRS) | 16.1 | 3.1 |  | | 16.4 | 4.2 |  | | 15.4 | 1.6 |  | |
| Number of medications | 5.9 | 2.1 |  | | 5.7 | 2.2 |  | | 5.4 | 1.6 |  | |
| Number of medication changes | 4.1 | 2.1 |  | | 4.0 | 1.8 |  | | 4.5 | 2.6 |  | |

No factors differed between responder and non-responder participants significantly. SD = standard deviation; MSM = Maudsley Staging Method, HDRS = Hamilton Depression Rating Scale; T1 = admission; T2 = discharge; CTQ = Childhood Trauma Questionnaire; MMSE = Mini Mental State Exam; MCIRS = Modified Cumulative Illness Rating Scale.


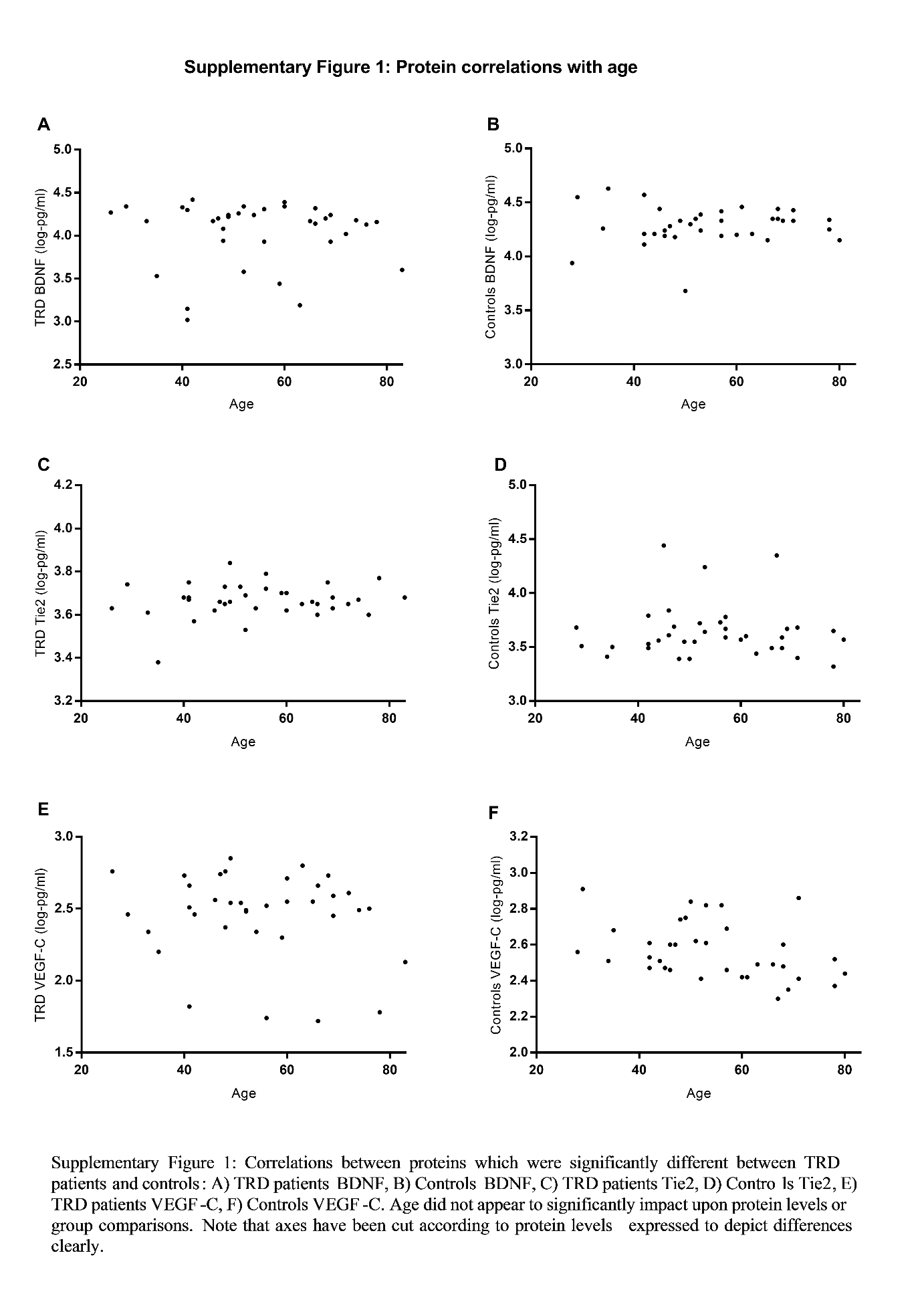

Supplement: Supplementary file 1 [file Data_Sheet_1.docx]
